# Consumption of low nutritive value foods and cardiometabolic risk factors among French-speaking adults from Quebec, Canada: the PREDISE study

**DOI:** 10.1186/s12937-019-0474-y

**Published:** 2019-08-29

**Authors:** Didier Brassard, Catherine Laramée, Véronique Provencher, Marie-Claude Vohl, Julie Robitaille, Simone Lemieux, Benoît Lamarche

**Affiliations:** 1 Centre Nutrition, santé et société (NUTRISS), Institute of Nutrition and Functional Food (INAF), Université Laval, Quebec City, Canada; 2 School of Nutrition, Université Laval, Quebec City, Canada

**Keywords:** 24-h recall, Diet quality, Usual intakes, Processed foods, Other foods, Web-based, Quebec, Canada, PREDISE, R24W

## Abstract

**Background:**

There is very limited knowledge on the magnitude to which foods with low nutritive value constitute the diet of adults from the province of Quebec. The extent to which these foods impact cardiometabolic risk is also poorly documented. The objective was to assess the contribution of low nutritive value foods to total energy intake (E) and to examine associations with cardiometabolic risk factors among French-speaking adults from 5 administrative regions of the Province of Quebec.

**Methods:**

As part of the cross-sectional PREDISE Study, 1147 adults (50.2% women; mean [SD] age, 43.2 [4.6] years) participated in a web-based investigation. Dietary intake data were obtained using a validated web-based self-administered 24-h recall, the R24W, completed on three occasions. Foods with low nutritive value were defined as foods exceeding predetermined thresholds for the following nutrients: saturated fat, sugar or sodium as well as beverages and ingredients not recommended in Canada’s Food Guide 2019. A total of 1019 participants underwent on-site clinical assessment of cardiometabolic risk factors.

**Results:**

Participants consumed on average 29.0%E (95%CI, 28.2–29.7) as low nutritive value foods, to which pastries (18%), alcohol (15%), sweets (13%), chips/popcorn (6%) and sugar-sweetened beverages (6%) contributed the most. Low nutritive value foods contributed more to total E in men than in women (30.7%E vs. 27.5%E, respectively; *P* < 0.0001). In fully-adjusted linear regression models, increments of 250 kcal/d from low nutritive value foods were associated with higher body mass index (+ 1.7 kg/m^2^; 95%CI 1.2 to 2.2), higher waist circumference (+ 0.6 cm; 95%CI, 0.1 to 1.1), cholesterol: HDL cholesterol ratio (+ 0.12 mmol/L; 95%CI, 0.01 to 0.24 and triglycerides (+ 7.8%; 95%CI, 3.0 to 12.8).

**Conclusions:**

Low nutritive value foods contribute near 30% of total daily energy intake of French-speaking adults of the Province of Quebec and are associated with increased waist circumference and an unfavourable lipid profile. Addressing consumption of low nutritive value foods at the population level is a potential strategy to attenuate the burden of chronic diseases.

**Electronic supplementary material:**

The online version of this article (10.1186/s12937-019-0474-y) contains supplementary material, which is available to authorized users.

## Introduction

Dietary habits, which are influenced by numerous individual, social and environmental factors [1], have a major role in determining health outcomes and chronic disease development [2]. This is why nutrition public health or agricultural policies are effective instruments for chronic disease prevention [3]. Knowledge of dietary intakes of the population is the cornerstone of such nutrition-focused public health policies [4]. Thus, monitoring dietary habits of the population is crucial for implementing impactful nutrition-focused public health policies aimed at chronic diseases prevention.

We have recently reported poor adherence of French-speaking adults from the Province of Quebec to 2007 dietary guidelines using the Canadian Healthy Eating Index (C-HEI) score [5]. A new Canada’s Food Guide (CFG) was just released in 2019 [6]. A key recommendation within these new guidelines is to limit the consumption of low nutritive value foods that contribute to excess sodium, free sugars or saturated fat intake [6]. Recent data on the consumption of such foods and their association with cardiometabolic health in the Canadian population are lacking.

As part of the **PREDISE** (***PRÉD****icteurs* ***I****ndividuels,* ***S****ociaux et* ***E****nvironnementaux*) study, dietary intakes of an age- and sex-representative sample of French-speaking adults from 5 administrative regions of the Province of Quebec were estimated using a validated, web-based 24-h recall instrument, the R24W [7–10]. The aims of this study were first to assess the contribution of low nutritive value foods to total energy intake (E) and, second, to examine associations with cardiometabolic risk factors. We hypothesized that low nutritive value foods 1) represent an important proportion of total energy intake in adults; 2) are associated with socio-economic status; and 3) are associated with a deteriorated cardiometabolic risk profile.

## Method

### Study design and participants

Data were collected as part of the multicenter cross-sectional PREDISE study, a web-based investigation designed to assess the association between individual, social and environmental factors and adherence to dietary guidelines among French-speaking adults from the Province of Quebec, Canada. Detailed methods have been reported elsewhere [5]. Briefly, adults were recruited by a survey firm via random digit dialing according to predetermined quotas based on five administrative regions (*Capitale-Nationale/Chaudière-Appalaches, Estrie, Mauricie, Montréal and Saguenay-Lac-St-Jean*), three age groups (18–34 y, 35–49 y, 50–65 y) and sex. The sampling quotas were defined according to age- and sex-specific proportions of French-speaking adults in each region. To be eligible, participants had to be between 18 and 65 years of age, to speak French as primary language at home, to have a computer, to have access to Internet and to have a valid email address. Pregnant and breastfeeding women were excluded. Participants that completed all study questionnaires were eligible for a draw to win a gift card or an electronic device. The study protocol was approved by the ethics board of each participating institution. The present study objectives were not pre-specified and are considered exploratory.

### Dietary assessment via web-based 24-h recall (the R24W)

#### The R24W

Participants completed the R24W on three unannounced random days over a period of 21 days. Details about the development and validation of the R24W have been reported elsewhere [7–10]. Briefly, the R24W is a self-administered web-based 24-h recall that has been validated in French-speaking adults using controlled feeding trials, i.e., objectively measured dietary intakes [7], and food records [8, 9]. Specifically, we have shown in controlled feeding studies that self-reported serving sizes from the R24W strongly correlated with serving sizes received with mean under-reporting of energy intakes that was smaller than 14 kcal [7]. The de-attenuated correlation coefficient (r = 0.49) for intake of low nutritive value foods between the R24W and food records as reference (9) is also within an acceptable range [11].

#### Low nutritive value foods

Low nutritive value foods refer to a broad range of foods, beverages and ingredients identified on the basis of thresholds for nutrients of public health concerns or foods not recommended in CFG-2019, as described in Table 1. CFG-2019 does not yet provide a clear definition of what low nutritive value foods entail, beyond the recommendation to limit consumption of highly processed products or of foods and ingredients contributing to saturated fats, sodium and sugars intake [6]. Therefore, nutrient thresholds used in this study were those proposed in the 2014 Health Canada Surveillance Tool (HCST) Tier System [12]. According to this classification, low nutritive value foods, termed Tier 4 foods, are those that exceed at least two predetermined thresholds for either total fat, saturated fats, sugars or sodium (Additional file 1: Table S1). Consistent with current guidelines, meeting only the total fat threshold does not classify a food as having a low nutritive value. Low nutritive value foods also include foods that are not recommended in CFG-2019, namely foods with a very high saturated/trans fat or sugar content and other ingredients and beverages described in Table 1. All mixed dishes in the R24W were disaggregated into their main components to identify those that meet the low nutritive value foods criteria (Additional file 1: Table S2). The energy provided by low nutritive value foods is calculated in the R24W based on the 2015 version of the Canadian Nutrient File [13]. As none of the food categories described in Table 1 are recommended in current dietary guidelines, their combined consumption was examined, and mean intakes were calculated using data from all available recalls.

**Table 1:** Description of low nutritive value foods

Description of low nutritive value foods	Examples in the R24W
1. Foods high in total fat, saturated fat, sugar or sodium^a^	Potato chips, some fries, croissant, ramen noodle, some cakes, some cookies, some cheese, some ice cream, breaded fish, fried chicken, some deli meats
*Exceed predetermined thresholds for at least two of the following criteria:*	
• *Total Fat: > 10 g per reference amount*	
• *Saturated fat: > 2 g per reference amount* ^b^	
• *Sugar: > 19 g per reference amount*	
• *Sodium: > 360 mg per reference amount*	
2. Foods with a very high saturated/trans fat content	Butter, coconut oil, shortening, cream
3. Foods with a very high sugar content	Sugar, honey, jam, syrup, candy, chocolate, fruit flavoured beverages
4. Other ingredients and beverages	Beer, wine, coffee, tea, ketchup, sauces, dipping

R24W, web-based 24-h recall

^a^ Nutrient thresholds and reference amounts were obtained in the 2014 Health Canada Surveillance Tool Tier System [12]. Meeting only the total fat threshold does not classify a food as having a low nutritive value

^b^ Foods from the milk and alternative or meat and alternatives food groups are not penalized for their saturated fat content under the 2014 Health Canada Surveillance Tool Tier System [12]

### Clinical assessment

Following the dietary assessment period, participants were invited for clinical assessment in their region-specific research center [5]. Height, weight, waist circumference and body fat percentage (BC-418, Tanita, Arlignton Heights, Il) were measured according to a standardized protocol. Office systolic and diastolic blood pressure were measured in the sitting position after a 10 min rest as the mean of three consecutive measurements taken 3 min apart (Digital BPM HEM-907XL model; Omron). For five participants, the assessment of blood pressure was based on only one or two measurements. Twelve-hour fasting blood samples were collected from an antecubital vein into evacuated tubes, centrifuged at 17 °C for 10 min at 1100×g and sera were stored at − 80 °C until serum total cholesterol, triglyceride, and high-density lipoprotein (HDL)-cholesterol concentrations assessment (Roche Modular P system, Roche Diagnostics, Mannheim, Germany). Serum low-density lipoprotein (LDL)-cholesterol concentrations were calculated with the use of the Friedewald equation. Fasting blood glucose concentrations were examined with the use of colorimetry (Hexokinase Method, Roche Modular P System), whereas insulin concentrations were tested with the use of electrochemiluminescence (Cobas 6000, Roche Diagnostics). HOMA-IR was calculated according to the method described by Matthews et al. [14]. All clinical data underwent double entry in the study management system. In participants that underwent clinical assessment, reasons for missing cardiometabolic risk markers included incomplete assessment (*n* = 3), loss of consciousness (*n* = 2) or unspecified (*n* = 15).

### Measurement error mitigation

#### Systematic error

Systematic error (or bias) such as under-reporting is more likely to occur with low nutritional value foods than with high nutritive value foods [15]. To mitigate this issue, the plausibility of reported energy intakes was assessed using the method by Huang et al. [16] according to which under- and over-reporters are those with a calculated energy intake:predicted energy requirement ratio < 0.78 and > 1.22, respectively (see Additional file 1: Table S3 and supplemental methods for details). Reporting status was included as a covariate in all analyses, as recommended elsewhere [17, 18]. We have assumed that data from the R24W provide unbiased estimates of population means once the plausibility of reported energy intakes has been considered.

#### Within-individual random error

Day-to-day and random variations in dietary intakes often result in attenuation of estimates of diet-outcome regression models [19]. Therefore, to account for within-individual random error, at least partially, we performed regression calibration using all R24W completed and the National Cancer Institute (NCI) methods 2.1 [19, 20]. Regression calibration is a technique where the “true” unobservable usual intakes are replaced by their best mean square error predictor [19]. In the present study, this corresponded to the conditional expectation of the low nutritive value foods intake, as estimated using data from each R24W available, and other covariates. Covariates for the NCI method to predict usual intake values were the same as those in the diet-outcome models and used as described elsewhere with the addition of sequence of recalls [19]. Since the majority of all recalls (98.9%) had reported intakes of low nutritive value foods greater than zero, the NCI’s “one-part amount model” was used. A SAS macro that performs regression calibration using the NCI’s “one-part amount model” is available online [21].

### Statistical analyses

**Fig. 1:**
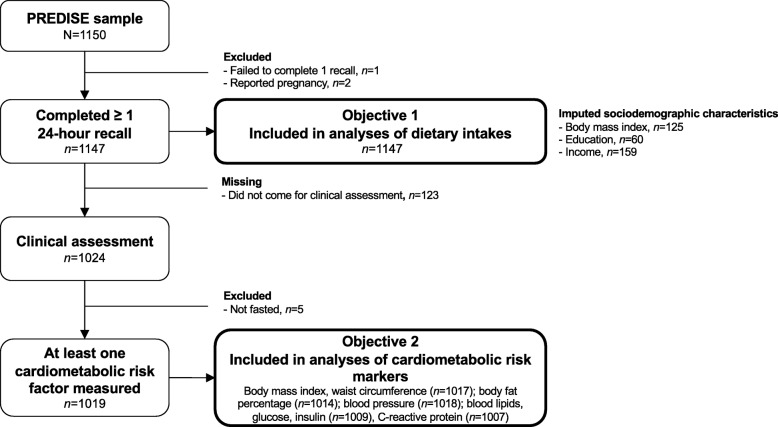
Participants’ flow chart in the PREDISE Study. Sample sizes are unweighted

The statistical software package SAS® Studio (v3.6, Cary, NC) and survey-specific procedures were used for all analyses. For the first objective, calories from low nutritive value foods were analyzed using absolute amounts (in kcal/d) as well as proportion of total energy intake (%E). Since the final PREDISE sample was larger than anticipated, the actual proportion of participants in each stratum slightly differed compared with what was planned originally [5]. To correct for these slight differences, sampling weights were used to ensure exact age- and sex-representativeness in each region [5]. Missing data for body mass index (BMI) category, education and income levels (Fig. 1) were imputed using the fully efficient fractional imputation methods, as detailed elsewhere [5]. Regression analyses were performed using the jackknife method for estimating variance, which considered the stratified design, sampling weights and the imputed sociodemographic characteristics. Least square means of subgroups were contrasted using PROC SURVEYREG and the Tukey-Kramer adjustment for multiple comparisons. Age group, sex, administrative region, BMI category, income and education levels, total number of weekend recalls and reporting status were included as covariates, when appropriate. The SURVEYMEANS procedure was used to obtain the mean contribution of specific food categories among low nutritive value foods to total energy intake.

For the second objective, multivariable regression analyses of the association between calories from low nutritive value foods (absolute amounts, in kcal/d) and cardiometabolic risk factors were performed using PROC SURVEYREG. Missing cardiometabolic risk factors were not imputed, because they are the dependent variables in these analyses. To address potential confounding, covariates considered in the models were as follows for *model 1*: sex, age, administrative region, total number of weekend recalls, reporting status (under-, plausible or over-reporter of total energy intake); *model 2*: model 1 and BMI (except for the outcome BMI), BMI-adjusted waist circumference (except for the outcome waist circumference and body fat percentage); *model 3*: model 2 and lifestyle-related factors including self-reported physical activity (0, 1–3, 4–6, 7 or unknown occurrence of moderate or intense physical activity sessions / week), dietary supplement usage (yes or no), medication usage (yes, no or unknown) and education level (high school or no diploma, CEGEP, university or unknown). The predicted usual intake values of calories from low nutritive value foods obtained using the NCI methods were used as a continuous exposure in the regression models to assess association for each increment of 250 kcal. This specific exposure was chosen a posteriori and corresponds roughly to half the interquartile range of reported intakes of low nutritive value foods. Not adjusting for energy intake was privileged since consumption of low nutritive value foods may inherently lead to overconsumption due to their high palatability and energy density [22]. However, we have further examined in sensitivity analyses associations of energy-adjusted exposure of low nutritive value foods to cardiometabolic risk markers. We also investigated the associations between calories from low nutritive value foods and cardiometabolic risk factor in participants stratified according to overall diet quality. Sex-specific medians of the C-HEI were used to dichotomize participants with an arbitrarily low or high diet quality.

Linear regression model fit was assessed visually using normal probability plots. Triglycerides (TG), blood glucose, insulin and HOMA-IR and C-reactive protein were log-transformed. As these are exploratory analyses, no family-wise error rate adjustments were performed.

## Results

### Flow chart and participants’ characteristics

A total of 1147 (objective 1) and 1019 (objective 2) participants were included in the analyses (Fig. 1). Their characteristics are presented in Table 2. Median [interquartile range] BMI was 26.3 [23.3–30.3] kg/m^2^. A total of 185 participants (16.2%) were considered likely to be under-reporters of energy intake. The group above the highest quartile of low nutritive value foods intake (as %E) included more men, younger participants, current smokers and potential over-reporters of energy intake, but fewer participants reporting dietary supplement usage (Table 2). Participants in the highest quarter of low nutritive value foods consumption reported greater intakes of saturated fats, total sugars and sodium (Table 2).

**Table 2:** Characteristics of 1147 French-speaking adults from 5 administrative regions of the Province of Quebec, Canada across level of exposure of low nutritive value foods

	Proportion of total energy intake from low nutritive value foods			
	Quarter 1 *n* = 286	Quarter 2 *n* = 286	Quarter 3 *n* = 287	Quarter 4 *n* = 287
	≤ 19.1%E	19.1–27.7%E	27.7–37.9%E	> 37.9%E
Mean dietary intake estimates/d				
Total energy intake, kcal	2169 (696)	2312 (648)	2457 (693)	2671 (830)
Saturated fats, g	27 (12)	30 (11)	34 (14)	38 (16)
Total sugars, g	100 (48)	110 (47)	115 (51)	127 (64)
Sodium, mg	3083 (1331)	3270 (1202)	3504 (1319)	3691 (1488)
Fibers, g	26 (12)	23 (8)	21 (8)	19 (8)
Vegetables and whole fruits, servings	5.7 (3.2)	4.9 (2.6)	4.5 (2.2)	3.5 (2.0)
Whole grain products, servings	2.2 (2.1)	1.6 (1.6)	1.4 (1.4)	1.0 (1.3)
Sex				
Men	19.8	26.2	24.4	29.5
Women	30.1	23.7	25.6	20.6
Age group, y				
18–34	23.6	22.8	23.7	29.9
35–49	27.0	27.5	22.2	23.3
50–65	24.7	25.0	28.7	21.6
Administrative region				
Estrie	24.0	28.2	26.5	21.3
Saguenay-Lac-Saint-Jean	22.7	24.5	20.6	32.2
Capitale-Nationale/Chaudière-Appalaches	24.4	23.7	25.5	26.4
Montreal	27.1	26.8	24.3	21.8
Mauricie	22.3	20.0	29.0	28.6
BMI group^a^				
Normal (< 25.0)	27.5	26.0	24.1	22.4
Overweight (25.0–29.9)	20.8	26.3	27.6	25.3
Obese (≥30.0)	26.3	21.9	23.2	28.6
Education^a^				
High school or no diploma	24.1	21.2	24.9	29.8
CEGEP ^c^	23.2	24.7	25.1	27.1
University	26.7	27.3	25.0	21.0
Household income^a^, $CAD				
< 30, 000	27.5	26.0	19.7	26.9
≥ 30, 000 to < 60, 000	28.1	21.3	25.4	25.2
≥ 60, 000 to < 90, 000	27.1	26.1	23.5	23.3
≥ 90, 000	20.0	26.9	28.1	25.0
Smoking				
Yes	17.6	18.5	23.3	40.7
Formerly	25.2	23.8	27.4	23.6
Never	26.8	27.4	24.0	21.7
Dietary supplement or health product usage				
Yes	33.1	20.8	26.4	19.7
No	22.0	26.5	24.5	27.0
Medication usage ^b^				
Yes	23.9	22.8	26.6	26.7
No	26.0	26.9	24.3	22.8
Occurrence of moderate or intense physical activity sessions / week ^b^				
None	24.4	22.7	26.7	26.1
1 to 3	23.0	26.5	25.7	24.8
4 to 6	22.9	23.3	27.6	26.2
7 or more	30.2	27.6	21.0	21.1
Reporting status				
Under-reporter (rEI:pER≤0.78)	39.0	26.1	16.4	18.5
Plausible reporter (0.78 < rEI:pER< 1.22)	24.2	26.0	25.7	24.2
Over-reporter (rEI:pER≥1.22)	18.9	22.5	28.4	30.1

Values are mean (SD) for dietary intakes and row percentage for demographic characteristics. All frequencies are weighted for exact age and sex-representativeness in each region. Rounding of weighted frequencies may have caused sample size to equal 1147 ± 1. Data were not further adjusted. *BMI* body mass index, *CEGEP* collège d’enseignement général et profesionnel, *CI* confidence intervals, *E* total energy intake, *pER* predicted energy requirements, *rEI* self-reported energy intake

^a^ Missing demographic characteristics have been imputed. See Material and methods for details

^b^ The numbers in subgroups may not sum to the total number of participants due to missing data. In such case, data were not imputed

^c^ CEGEP is a pre-university and technical college institution specific to the Quebec educational system

### Low nutritive value foods consumption

**Fig. 2:**
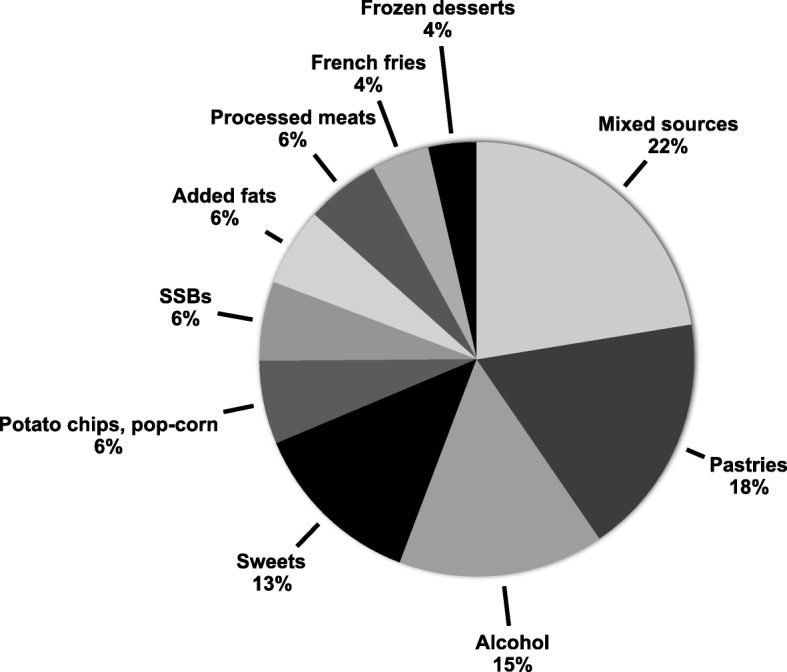
Relative contribution of individual foods to the average 723 kcal consumed daily as low nutritive value foods in 1147 French-speaking adults from Quebec. SSBs include fruit-flavored drinks, sodas, sport drinks, energy drinks, sweetened coffee or tea. Pastries include sugary bars, muffins and breads, donuts, cookies, crunchies, bun, croissant, cake, pie, pastries. Sweets include candies, chocolate, jam, honey, syrup, molasse. Added fats include cream, butter and shortening among others. Mixed sources represent all other sources, for example condiments, fried foods and supplemented foods. CFG, Canada’s Food Guide; E, total energy intake; SSBs, sugar-sweetened beverages

**Fig. 3:**
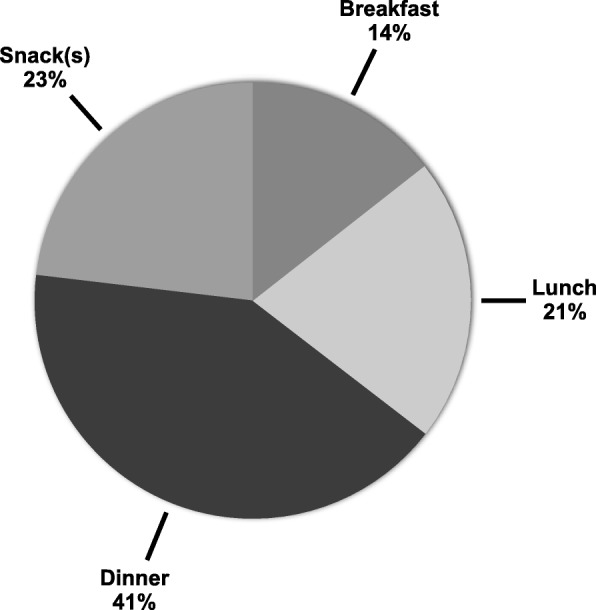
Relative distribution at each meal of the average 723 kcal consumed daily as low nutritive value foods in 1147 French-speaking adults from Quebec

French-speaking adults from Quebec consumed on average 723 kcal per day (95%CI, 697–749 kcal) from low nutritive value foods, which corresponded to 29.0% (95%CI, 28.2–29.7) of total energy intake (Table 3). Median intake (27.7%E) was slightly lower than mean intake (Additional file 1: Table S4). Intake of low nutritive value foods contributed to a greater proportion of daily energy intake in men than in women (mean difference, 3.2%E; *P* < 0.0001). Compared with participants in the 35–49 y and 50–65 y age categories, those in the 18–34 y category consumed an additional 2.9%E (*P* = 0.009) and 3.4%E (*P* = 0.0004) as low nutritive value foods, respectively. BMI was positively while education level was negatively associated with the %E from low nutritive value foods. Pastries (18%), alcohol (15%), sweets (13%), potato chips/popcorn (6%) and sugar sweetened beverages (6%) were the main single food sources contributing to the 723 daily kcal as low nutritive value foods (Fig. 2). The proportion of total energy intake from single sources of low nutritive value foods according to sociodemographic subgroups is presented in Additional file 1: Figure S1. The majority of the daily calories consumed as low nutritive value foods (41%) was consumed at dinner (Fig. 3). The pattern of consumption at different meals was similar among the various sociodemographic subgroups (not shown).

**Table 3:** Multivariable least square means of low nutritive value foods intakes in 1147 French-speaking adults from 5 administrative regions of the Province of Quebec, Canada

Sociodemographic characteristics	*n* (weighted)	Intake of low nutritive value foods, mean (95%CI)	
		Kcal/d	% of total energy
All			
All	1147	723 (697–749)	29.0 (28.2–29.7)
Sex			
Men	571	838 (792–884) ^a^	30.7 (29.3–32.2) ^a^
Women	576	572 (537–608) ^b^	27.5 (26.2–28.9) ^b^
*P*		< 0.0001	< 0.0001
Age group, y			
18–34	408	799 (749–849) ^a^	31.2 (29.6–32.8) ^a^
35–49	338	694 (644–743) ^b^	28.3 (26.6–30.1) ^b^
50–65	400	622 (582–663) ^c^	27.8 (26.4–29.3) ^b^
*P*		< 0.0001	0.0004
BMI group^a^			
Normal (< 25.0)	453	611 (569–653) ^a^	27.6 (26.1–29.2) ^a^
Overweight (25.0–29.9)	383	694 (647–742) ^b^	29.1 (27.5–30.7) ^a,b^
Obese (≥30.0)	312	810 (759–861) ^c^	30.6 (29.0–32.3) ^b^
*P*		< 0.0001	0.006
Education^a^			
High school or no diploma	284	734 (683–786)	30.2 (28.4–31.9) ^a^
CEGEP ^b^	353	712 (666–759)	29.5 (28.0–31.1) ^a,b^
University	510	669 (624–713)	27.7 (26.1–29.3) ^b^
*P*		0.06	0.03
Household income^a^, $CAD			
< 30, 000	192	731 (663–798) ^a,b^	29.6 (27.5–31.7)
≥ 30, 000 to < 60, 000	328	705 (657–752) ^a,b^	28.7 (27.1–30.3)
≥ 60, 000 to < 90, 000	227	652 (603–701) ^a^	27.9 (26.1–29.7)
≥ 90, 000	400	733 (688–778) ^b^	30.3 (28.8–31.8)
*P*		0.02	0.07

All values are mean (95%CI). *P*-values are the partial effect of the sociodemographic characteristics on intake in the linear models. Except for the “all” estimates, subgroup least square means are adjusted for age group, sex, administrative region, BMI group, education and household income level, reporting status and the number of weekend recalls, when appropriate. Subgroups least square means without a common superscript letter indicate a low p-value (*P* < 0.05, Tukey-Kramer). Rounding of weighted frequencies may have caused sample size to equal 1147 ± 1

*BMI* body mass index, *CI* confidence intervals

^a^ Missing characteristics have been imputed. See Material and methods for details

^b^ CEGEP is a pre-university and technical college institution specific to the Quebec educational system

### Cardiometabolic risk

Table 4 depicts the variation in cardiometabolic risk factors for each increment of 250 kcal from low nutritive value foods. In fully-adjusted linear regression models, each increment of 250 kcal from low nutritive value foods was associated with a greater waist circumference (+ 0.6 cm; 95%CI 0.1–1.1) and BMI (+ 1.7 kg/m^2^; 95%CI 1.2–2.2). Low nutritive value foods consumption was also associated with a greater concentration of most serum lipid concentrations. Notably, an increment of 250 cal from low nutritive value foods was associated with higher serum total cholesterol (+ 0.11 mmol/L; 95%CI 0.01–0.21), non-HDL cholesterol (+ 0.12 mmol/L; 95%CI 0.03–0.22) and TG (+ 7.8%; 95%CI, 3.0–12.8%) and with a higher cholesterol: HDL cholesterol ratio (+ 0.12; 95%CI 0.01 to 0.24).

**Table 4:** Associations between incremental consumption of 250 cal from low nutritive value foods and cardiometabolic risk factors

	Regression coefficients (95%CI)		
	Model 1	Model 2	Model 3
Anthropometric measures			
Waist circumference, cm	4.8 (3.6 to 6.0)	0.8 (0.3 to 1.3)	0.6 (0.1 to 1.1)
*P*	< 0.001	0.002	0.02
BMI, kg/m^2^	1.8 (1.4 to 2.3)	1.9 (1.4 to 2.4)	1.7 (1.2 to 2.2)
*P*	< 0.001	< 0.001	< 0.001
Body fat, %	2.2 (1.6 to 2.8)	0.2 (−0.1 to 0.5)	0.1 (−0.2 to 0.5)
*P*	< 0.001	0.13	0.38
Serum lipids, mmol/L			
Total cholesterol	0.09 (0.01 to 0.18)	0.11 (0.01 to 0.20)	0.11 (0.01 to 0.21)
*P*	0.03	0.02	0.03
LDL cholesterol	0.06 (−0.01 to 0.13)	0.05 (− 0.03 to 0.13)	0.06 (− 0.02 to 0.15)
*P*	0.10	0.19	0.15
HDL cholesterol	−0.06 (− 0.09 to − 0.02)	− 0.01 (− 0.05 to 0.03)	−0.01 (− 0.05 to 0.03)
*P*	0.002	0.54	0.62
TG^a^, %	13.7 (9.1 to 18.5)	9.0 (4.3 to 13.8)	7.8 (3.0 to 12.8)
*P*	< 0.001	< 0.001	0.001
Non-HDL cholesterol	0.15 (0.07 to 0.23)	0.12 (0.03 to 0.20)	0.12 (0.03 to 0.22)
*P*	< 0.001	0.01	0.01
Cholesterol:HDL cholesterol	0.23 (0.13 to 0.33)	0.12 (0.02 to 0.23)	0.12 (0.01 to 0.24)
*P*	< 0.001	0.03	0.04
Blood pressure, mm Hg			
SBP	1.7 (0.6 to 2.7)	0.2 (−0.8 to 1.3)	0.2 (−0.9 to 1.3)
*P*	0.002	0.66	0.73
DBP	1.7 (0.8 to 2.5)	0.5 (−0.4 to 1.4)	0.4 (−0.5 to 1.3)
*P*	< 0.001	0.26	0.37
Glucose homeostasis			
Fasting glucose^a^, %	0.3 (−0.7 to 1.3)	− 0.9 (−2.0 to 0.3)	−0.9 (−2.1 to 0.3)
*P*	0.60	0.14	0.15
Fasting insulin^a^, %	8.0 (3.9 to 12.2)	0.2 (−3.6 to 4.1)	−0.3 (−4.2 to 3.8)
*P*	< 0.001	0.94	0.87
HOMA-IR^a^, %	8.3 (3.7 to 13.0)	−0.7 (− 4.9 to 3.6)	−1.2 (−5.5 to 3.3)
*P*	< 0.001	0.74	0.59
Inflammation			
C-reactive protein^a^, %	28.0 (16.4 to 40.8)	3.5 (−4.9 to 12.6)	2.4 (−6.2 to 11.7)
*P*	< 0.001	0.43	0.60

All values are regression coefficients (95% CI). P-values are the partial effect of calories from low nutritive value foods in the linear models. Usual intakes of low nutritive value foods were computed using the NCI method 2.1 and one-part models. Covariates are as follows

Model 1: age, sex, center, number of weekend recalls, reporting status

Model 2: model 1 and BMI (except for the outcome BMI), BMI-adjusted waist circumference (except for the outcome waist circumference and body fat percentage)

Model 3: model 2 and physical activity, smoking, dietary supplement usage, medication usage and education level

*BMI* body mass index, *DBP* diastolic blood pressure, *CI* confidence intervals, *HDL* high-density lipoproteins, *LDL* low-density lipoproteins, *SBP* systolic blood pressure, *TG* triglycerides

^a^ Analyses were performed on log-transformed data. Hence, values are expressed as percentage change upon backtransformation calculated as 100 × exponential (logβ_x_) – 100

Assessing these associations without correction for within-person random error (increment of 250 kcal/d of low nutritive value foods, Additional file 1: Table S5**;** increment of 10%E from low nutritive value foods, Additional file 1: Table S6) yielded estimates that were generally consistent to those seen when the within-person random error is accounted for. Further adjustment for diet quality and total energy intake also did not materially modify the magnitude of the associations between intake of low nutritive quality foods and cardiometabolic risk factors (Additional file 1: Table S5). The only exception was the attenuation of the association with BMI. Finally, in participants with an arbitrarily high overall diet quality compared to those with a diet of lower quality, an increment of 250 kcal/d from low nutritive value foods was less strongly associated with waist circumference (+ 0.2 vs. 1.0 cm, respectively), BMI (+ 1.0 vs 2.8 kg/m^2^, respectively) and triglycerides (+ 4.6% vs. 7.5%, respectively, Additional file 1: Table S7).

## Discussion

In this study, we used for the first time in Canada a validated web-based 24-h recall, the R24W, to report population-level intake estimates of low nutritive value foods. Among this age- and sex-representative sample of French-speaking adults of 5 regions of the Province of Quebec, we found that low nutritive value foods contributed to a nearly a third of total energy intake. This proportion was greater in men than in women, in participants with obesity and in participants with a lower education degree. Consistent with our hypothesis, greater intake from low nutritive value foods was associated with an adverse cardiometabolic risk profile including increased waist circumference, BMI and a perturbed serum lipid profile. Our data reinforce the importance of strong public health actions targeting foods with low nutritional value to impact the health of the population in Quebec and Canada, such as supporting food reformulation to improve nutritional quality of food.

The 2014 HCST Tier System [12] was used in the present study to identify some low nutritive value foods. This nutrient profiling method has been criticized due to its inability to predict the risk of obesity in Canadian adults surveyed in CCHS 2004 [23] and its lack of consideration of food processing [24]. Indeed, we must acknowledge that the classification used in the present study for low nutritive value foods does not explicitly consider the degree of food processing, which may also provide information on food and diet quality [22, 24, 25]. On the other hand, the recently released Food Guide in Canada defines poor quality foods as “processed or prepared foods and beverages that contribute to excess sodium, free sugars, or saturated fat when consumed on a regular basis” [6]. These guidelines rely to a large extent on nutrient profiling to characterize low nutritive value foods rather than on food processing per se.

Comprehensive food and nutrient intake analyses derived from the most recent national survey (CCHS 2015) in Canada have yet to be published. Therefore, the most recent estimates of low nutritive value foods consumption in Canadians are derived from CCHS 2004 data. Blanchet et al. [26] reported dietary intakes specific to adults of the Province of Quebec using CCHS 2004 data. In their report, low nutritive value foods were the “other foods” category in CFG 1992 and contributed to 24.2%E (95%CI, 23.0 to 25.3) of the diet of adults of the province of Quebec [26]. In the same report, the main sources of “other foods” in adults from Quebec were sweets and desserts, alcohol and sugar-sweetened beverages. More recently, Jessri et al. [23] described dietary intakes of Canadian adults using data from CCHS 2004 and the HCST Tier system. Consumption of low nutritive value foods, also termed “other foods”, ranged from 23%E to 31%E according to age (19 to 70 y) and sex. Low nutritive value foods consumption in adults from Quebec as reported by Blanchet et al. [26] is lower than in the present study (i.e., 29%E). On the other hand, we also found that pastries, alcohol, sweets and sugar sweetened beverages were the major single foods contributing to total intake of low nutritive value foods in the diet of adults from Quebec, consistent with data from Blanchet et al. [26]. Estimates of overall contribution of low nutritive value foods to total energy intake in Canadian adults obtained by Jessri et al. [23] are more consistent with our data, which is unsurprising because both studies used a similar definition of low nutritive value foods. Finally, the 24-h recall was interviewer-administered in CCHS 2004 and self-administered on the Web in the present study. Nevertheless, data suggest that consumption of lower nutritive value foods has barely changed over the years, at least in French-speaking adults from Quebec.

Low nutritive value foods are by definition dense in saturated fat, sugar or sodium which, in turn, have been shown to influence cardiometabolic risk markers. A meta-analysis of 39 controlled trials revealed that higher compared with lower intakes of free sugars increased total and LDL cholesterol, TG and blood pressure [27]. However, another meta-analysis of isoenergetic intervention trials revealed unclear effects of free sugars on blood lipids when substituted for complex carbohydrates [28]. SSBs in particular are important contributors to weight gain, type 2 diabetes and cardiovascular disease risks [29]. A meta-analysis of trials comparing effects of fat and oils on blood lipids demonstrated that saturated fat, especially in the form of butter or lard, increased LDL cholesterol more than most unsaturated fat oils [30]. A meta-analysis of interventional studies revealed that heavy alcohol consumption (greater than 60 g per day), but not moderate, increased triglyceride concentrations [31]. A reduction in alcohol consumption lowers systolic and diastolic blood pressure according to another meta-analysis of randomized controlled trials [32]. In the present study, the combination of saturated fats, sugars, sodium provided by low nutritive value foods may have accentuated associations with cardiometabolic markers compared with associations based on one nutrient only. Associations of calories from low nutritive value foods with systolic and diastolic blood pressure, fasting insulin, HOMA-IR and C-reactive protein were attenuated when adiposity was accounted for in the models. Calories from low nutritive value foods may nevertheless have an indirect effect on these risk factors considering their association with BMI and waist circumference.

This cross-sectional study has several strengths including the use of a validated web-based 24-h recall on repeated occasions, consideration of systematic error and within-person random errors in the analyses, an objective and accepted definition of low nutritive value foods and a comprehensive assessment of cardiometabolic risk markers. Limitations must also be acknowledged. Although associations between consumption of low nutritive value foods and cardiometabolic risk profile are consistent with data from randomized controlled trials [27, 30, 31], the possibility of unaccounted residual confounding cannot be excluded. Second, the cross-sectional design of the PREDISE study limits inference regarding causality. For example, sensitivity analyses revealed that some associations such as the ones between BMI, waist circumference, triglycerides and calories from low nutritive value foods are modulated by overall diet quality, at least to some extent. As type I errors are probable under such design and multiple hypothesis testing, *p* values must be interpreted with caution. Third, multivariate modeling of correlated dietary components that are each subject to random error is an area of ongoing work [33]. Associations between usual intakes of low nutritive value foods and cardiometabolic risk could not be adjusted directly for concurrent variations in C-HEI, the proxy of diet quality. The fact that the associations between intake of low nutritive value foods and cardiometabolic risk factors appear to be independent of the C-HEI and total energy intake needs to be interpreted with caution. Indeed, such analysis of highly correlated variables that are not adjusted for within-person random error has the potential to produce spurious results. Fourth, the assumption that dietary intake estimates derived from the R24W are unbiased is unlikely to be satisfied, even when considering the plausibility of reported energy intakes. Fifth, despite the fact that participants in the PREDISE study are representative of the age and sex population among French-speaking adults from the 5 administrative regions investigated, individuals with post-secondary education are over-represented, as described elsewhere [5]. This suggests that the true contribution of low nutritive value foods to total energy intake in the Quebec population may be even greater than what we have reported here. Finally, an updated version of the 2014 HCST is expected in the future to match the update of CFG [6]. Although the classification of some foods may change, the definition of low nutritive value foods in the present study is mostly based on saturated fats, sodium and sugars thresholds, which are consistent with the most recent CFG 2019 recommendations. Nonetheless, future analyses of low nutritive value food consumption will need to account for variations in the definition and classification of these foods.

## Conclusion

In sum, in spite of long-standing recommendations to limit consumption of foods, beverages and ingredients high in saturated fat, sugar or sodium, low nutritive value foods rich in these nutrients contribute to nearly one third of total daily energy intake among French-speaking adults from 5 regions of Province of Quebec. Our data further support the need to address the importance of low nutritive value foods and the resulting low quality diet in this population [5], especially in light of clinically meaningful associations between consumption of such foods and increased waist circumference as well as a unfavourable lipid profile. Monitoring dietary intake over time, with consideration of both nutritive and less nutritive foods, will help to determine the effectiveness of nutrition-focused public health policies.

## Additional file

Additional file 1: **Table S1.** Classification of some foods high in saturated fats, sugar or sodium in the R24W using the Health Canada Surveillance Tool Tier System. **Table S2.** Examples of calories from low nutritive value foods in mixed dishes as calculated in the R24W. **Table S3.** Estimation of standard deviation stratified by BMI, sex and age group among participants that came for clinical assessment. **Table S4.** Median intakes of low nutritive value foods among 1147 French-speaking adults from 5 administrative regions of the Province of Quebec, Canada. **Table S5.** “Naive” associations between incremental consumption of 250 cal from low nutritive value foods and cardiometabolic risk markers. **Table S6.** “Naive” associations between incremental consumption of 10% of total energy intakes from low nutritive value foods and cardiometabolic risk markers. **Table S7.** Associations between incremental consumption of 250 cal from low nutritive value foods and cardiometabolic risk markers, stratified by median Canadian Healthy Eating Index scores. **Figure S1.** Proportion of total energy intake provided by the main sources of low nutritive value foods according to sociodemographic characteristics. (PDF 337 kb)

## Data Availability

The datasets used in this study are available from the corresponding author on reasonable request.

## Abbreviations

BMI: Body mass index
CCHS: Canadian Community Health Survey
CFG: Canada’s Food Guide
C-HEI: Canadian Healthy Eating Index
HCST: Health Canada Surveillance Tool
HDL: High-density lipoprotein
LDL: Low-density lipoprotein
NCI: National Cancer Institute
R24W: Web-based 24-h recall
TG: Triglycerides

## References

[1] Mozaffarian D (2016). Dietary and policy priorities for cardiovascular disease, diabetes, and obesity: a comprehensive review. Circulation..

[2] Alam S, Lang JJ, Drucker AM, Gotay C, Kozloff N, Mate K (2019). Assessment of the burden of diseases and injuries attributable to risk factors in Canada from 1990 to 2016: an analysis of the global burden of disease study. CMAJ Open.

[3] Yu E, Malik VS, Hu FB (2018). Cardiovascular disease prevention by diet modification: JACC health promotion series. J Am Coll Cardiol.

[4] Labonté M, Kirkpatrick SI, Bell RC, Boucher BA, Csizmadi I, Koushik A, et al. Dietary assessment is a critical element of health research - perspective from the Partnership for Advancing Nutritional and Dietary Assessment in Canada. Appl Physiol Nutr Metab. 2016:1–4. 10.1139/apnm-2016-0146 27608060

[5] Brassard D, Laramée C, Corneau L, Bégin C, Bélanger M, Bouchard L (2018). Poor adherence to dietary guidelines among French-speaking adults in the province of Quebec Canada: the PREDISE study. Can J Cardiol.

[6] Health Canada. Canada’s food guide 2019 [Available from: https://food-guide.canada.ca/en/.

[7] Lafrenière J, Lamarche B, Laramée C, Robitaille J, Lemieux S (2017). Validation of a newly automated web-based 24-hour dietary recall using fully controlled feeding studies. BMC Nutrition.

[8] Lafrenière J, Laramée C, Robitaille J, Lamarche B, Lemieux S (2018). Assessing the relative validity of a new, web-based, self-administered 24 h dietary recall in a French-Canadian population. Public Health Nutr.

[9] Lafreniere J, Laramee C, Robitaille J, Lamarche B, Lemieux S (2018). Relative validity of a web-based, self-administered, 24-h dietary recall to evaluate adherence to Canadian dietary guidelines. Nutrition..

[10] Jacques S, Lemieux S, Lamarche B, Laramée C, Corneau L, Lapointe A (2016). Development of a web-based 24-h dietary recall for a French-Canadian population. Nutrients..

[11] Willett W (2013). Nutritional epidemiology.

[12] Health Canada. The development and use of a surveillance tool the classification of foods in the Canadian nutrient file according to Eating well with Canada’s food guide [PDF]. Ottawa 2014 [1–25]. Available from: http://publications.gc.ca/collections/collection_2014/sc-hc/H164-158-2-2014-eng.pdf.

[13] Health Canada. Canadian Nutrient File (CNF). 2015 [Available from: https://aliments-nutrition.canada.ca/cnf-fce/.

[14] Matthews DR, Hosker JP, Rudenski AS, Naylor BA, Treacher DF, Turner RC (1985). Homeostasis model assessment: insulin resistance and beta-cell function from fasting plasma glucose and insulin concentrations in man. Diabetologia..

[15] Livingstone MB, Black AE (2003). Markers of the validity of reported energy intake. J Nutr.

[16] Huang TT, Roberts SB, Howarth NC, McCrory MA (2005). Effect of screening out implausible energy intake reports on relationships between diet and BMI. Obes Res.

[17] Jessri M, Lou WY, L'Abbé MR (2016). Evaluation of different methods to handle misreporting in obesity research: evidence from the Canadian national nutrition survey. Br J Nutr.

[18] Tooze JA, Freedman LS, Carroll RJ, Midthune D, Kipnis V (2016). The impact of stratification by implausible energy reporting status on estimates of diet-health relationships. Biom J.

[19] Kipnis V, Midthune D, Buckman DW, Dodd KW, Guenther PM, Krebs-Smith SM (2009). Modeling data with excess zeros and measurement error: application to evaluating relationships between episodically consumed foods and health outcomes. Biometrics..

[20] Tooze JA, Midthune D, Dodd KW, Freedman LS, Krebs-Smith SM, Subar AF (2006). A new statistical method for estimating the usual intake of episodically consumed foods with application to their distribution. J Am Diet Assoc.

[21] National Cancer Institute. Usual dietary intakes: SAS macros for analysis of a single dietary component [Available from: https://epi.grants.cancer.gov/diet/usualintakes/macros_single.html.

[22] Hall KD, Ayuketah A, Brychta R, Cai H, Cassimatis T, Chen KY, et al. Ultra-processed diets cause excess calorie intake and weight gain: an inpatient randomized controlled trial of ad libitum food intake. Cell Metab. 2019;30(1):226. 10.1016/j.cmet.2019.05.020 PMC7959109 31269427

[23] Jessri M, Nishi SK, L'Abbé MR (2015). Assessing the nutritional quality of diets of Canadian adults using the 2014 Health Canada surveillance tool tier system. Nutrients..

[24] Moubarac JC, Batal M, Louzada ML, Martinez Steele E, Monteiro CA (2017). Consumption of ultra-processed foods predicts diet quality in Canada. Appetite..

[25] Nardocci M, Leclerc BS, Louzada ML, Monteiro CA, Batal M, Moubarac JC (2019). Consumption of ultra-processed foods and obesity in Canada. Can J Public Health.

[26] Blanchet C, Plante C, Rochette L (2009). Institut national de santé publique du Québec. La consommation alimentaire et les apports nutritionnels des adultes québécois.

[27] Te Morenga LA, Howatson AJ, Jones RM, Mann J (2014). Dietary sugars and cardiometabolic risk: systematic review and meta-analyses of randomized controlled trials of the effects on blood pressure and lipids. Am J Clin Nutr.

[28] Fattore E, Botta F, Agostoni C, Bosetti C (2017). Effects of free sugars on blood pressure and lipids: a systematic review and meta-analysis of nutritional isoenergetic intervention trials. Am J Clin Nutr.

[29] Malik VS, Popkin BM, Bray GA, Despres JP, Hu FB (2010). Sugar-sweetened beverages, obesity, type 2 diabetes mellitus, and cardiovascular disease risk. Circulation..

[30] Schwingshackl L, Bogensberger B, Bencic A, Knuppel S, Boeing H, Hoffmann G (2018). Effects of oils and solid fats on blood lipids: a systematic review and network meta-analysis. J Lipid Res.

[31] Brien SE, Ronksley PE, Turner BJ, Mukamal KJ, Ghali WA (2011). Effect of alcohol consumption on biological markers associated with risk of coronary heart disease: systematic review and meta-analysis of interventional studies. BMJ..

[32] Roerecke M, Kaczorowski J, Tobe SW, Gmel G, Hasan OSM, Rehm J (2017). The effect of a reduction in alcohol consumption on blood pressure: a systematic review and meta-analysis. Lancet Public Health.

[33] Kipnis V, Freedman LS, Carroll RJ, Midthune D (2016). A bivariate measurement error model for semicontinuous and continuous variables: application to nutritional epidemiology. Biometrics..

