# Supplementary material for: Consumption of low nutritive value foods and cardiometabolic risk factors among French-speaking adults from Quebec, Canada: the PREDISE study
Source: Nutr J. 2019 Aug 29;18:49. doi: 10.1186/s12937-019-0474-y (PMC6716857; doi:10.1186/s12937-019-0474-y)
Supplement: Supplementary file 1 — Table S1. Classification of some foods high in saturated fats, sugar or sodium in the R24W using the Health Canada Surveillance Tool Tier System. Table S2. Examples of calories from low nutritive value foods in mixed dishes as calculated in the R24W. Table S3. Estimation of standard deviation stratified by BMI, sex and age group among participants that came for clinical assessment. Table S4. Median intakes of low nutritive value foods among 1147 French-speaking adults from 5 administrative regions of the Province of Quebec, Canada. Table S5. “Naive” associations between incremental consumption of 250 cal from low nutritive value foods and cardiometabolic risk markers. Table S6. “Naive” associations between incremental consumption of 10% of total energy intakes from low nutritive value foods and cardiometabolic risk markers. Table S7. Associations between incremental consumption of 250 cal from low nutritive value foods and cardiometabolic risk markers, stratified by median Canadian Healthy Eating Index scores. Figure S1. Proportion of total energy intake provided by the main sources of low nutritive value foods according to sociodemographic characteristics. (PDF 337 kb) [file 12937_2019_474_MOESM1_ESM.pdf]

## Supplemental methods

### 1. Definition of low nutritive value foods

**Supplemental Table S1.** Classification of some foods high in saturated fats, sugar or sodium in the R24W using the Health Canada Surveillance Tool Tier System

| Type of food | Tier 4 foods (i.e. exceeding at least two predetermined nutrient thresholds*) | Not considered as low nutritive value foods |
|---|---|---|
| **Vegetables and fruit** | | |
| French fries | - Cooked in oil<br>- Snack bar/restaurant | -Baked |
| **Grain products** | | |
| Cookies | - Chocolate chip<br>- Chocolate covered<br>- Macaron<br>- Oatmeal, commercial<br>- Filled with cream<br>- Shortbread | -Fig, date, fruit<br>-Ginger<br>-Molasses<br>-Oatmeal, homemade<br>-Plain (e.g. Social Tea)<br>-Wafer |
| Cake | - Carrot<br>- Cheese<br>- Chocolate<br>- Upside-down (e.g. pineapple, pear, apple)<br>- White vanilla | -Angel |
| Pop-corn | - With butter | -Plain |
| **Milk and alternatives** | | |
| Ice cream | - Regular | - Low fat<br>- Without added sugar |
| Cheese | - Processed, regular<br>- Cream | - Processed, low fat |
| **Meat and alternatives** | | |
| Deli meat | -Bologna<br>-Salami or peperoni<br>-Prosciutto<br>-Bacon<br>-Sausage | -Ham, sliced beef, turkey or chicken breast |

* Nutrient thresholds and reference amounts were obtained in the 2014 Health Canada Surveillance Tool Tier System (1). Meeting only the total fat threshold does not classify a food as having a low nutritive value.
R24W, web-based 24-hour recall.

## Supplemental methods

**Supplemental Table S2**. Examples of calories from low nutritive value foods in mixed dishes as calculated in the R24W

| Mixed dishes | Reference amount (g) | Total energy from low nutritive value foods per reference amount (kcal) | Food source of energy considered as low nutritive value foods |
|---|---|---|---|
| All-dressed pizza | 200 | 75 | Pepperoni |
| Burger with cheese* | 200 | 77 | Processed cheese and ketchup |
| Chicken club sandwich | 200 | 55 | Bacon |
| Chicken Pad Thai | 300 | 10 | Chili sauce and fish sauce |
| Ham quiche | 200 | 163 | Pie crust |
| Legume curry | 300 | 278 | Honey and coconut milk |

*For example, a burger would be divided as grain products (i.e., bread), meat and alternatives (i.e., beef), vegetables (i.e., lettuce and tomato) and low nutritive value foods (i.e., processed cheese and ketchup).
R24W, web-based 24-hour recall.

## Supplemental methods

### 2. Plausibility of self-reported energy intakes using the R24W

Identification of the plausibility of self-reported energy intake (rEI) was conducted using the method proposed by Huang *et al.* (2). Predicted energy requirements (pER) were calculated using the equations provided by the *Institute of Medecine* (3). Furthermore, to calculate predicted energy requirements in participants who did not come on-site for clinical assessment (*n*=123) or that had missing height and weight measurements (*n*=2), missing height and weight – roughly 11% of data – were imputed using the hot-deck imputation method in SAS®'s SURVEYIMPUTE procedure. Missing values were treated as if they were observed values, since a robust variance estimation for height and weight is not required in the equation below.
An objective measure of physical activity is lacking in the present study. Therefore, all subjects were assumed to have a sedentary level of activity. The latter assumption may overestimate the true proportion of over-reporters but is more sensitive to identify true under-reporters.
Participants were classified as under-reporters, plausible reporters or over-reporters on the basis of their ratio of rEI:pER, i.e. a ratio of 1.00 would indicate exact correspondence between both measures. Confidence limits were calculated around the rEI:pER ratio to account for measurement errors and normal variation in energy expenditure (**Supplemental Table S3**):

$$\pm 1\ SD = \sqrt{\frac{CV_{rEI}^2}{d} + CV_{pER}^2 + CV_{mTEE}^2}$$

where $CV_{rEI}$ is the within-individual variation in rEI among the 24-hour recalls completed (i.e., 29.2%) and d is the number of 24-hour recall completed (i.e., 3 days). The $CV_{rEI}$ was determined using MIXED models in SAS® with participant as random effect and 24-hour recall as fixed effect. $CV_{pER}$ is the within-individual variation in predicted energy requirements (i.e., 10.9%) and $CV_{mTEE}$ is the normal day-to-day variation and error associated with objective measurement of total energy expenditure (i.e., 8.2%), as detailed elsewhere (4). Thus, under- and over-reporter had a self-reported energy intake to predicted energy requirements ratio < 0.78 and > 1.22, respectively.

## Supplemental methods

**Supplemental Table S3**. Estimation of standard deviation stratified by BMI, sex and age group among participants that came for clinical assessment

| Categories | *n* | $CV_{rEI}$ | $CV_{pER}$ | $CV_{mTEE}$ | ±1 SD |
|---|---|---|---|---|---|
| **All** | 1022 | 29.2 | 10.9 | 8.2 | 21.7% |
| **BMI <25 kg/m²** | | | | | |
| **Men** | | | | | |
| 18-34 | 88 | 39.7 | 6.7 | 8.2 | 25.2% |
| 35-49 | 42 | 25.0 | 6.6 | 8.2 | 17.9% |
| 50-65 | 40 | 20.2 | 5.6 | 8.2 | 15.3% |
| **Women** | | | | | |
| 18-34 | 89 | 24.6 | 5.8 | 8.2 | 17.4% |
| 35-49 | 64 | 26.8 | 5.2 | 8.2 | 18.3% |
| 50-65 | 84 | 26.1 | 6.0 | 8.2 | 18.2% |
| **BMI ≥25 kg/m²** | | | | | |
| **Men** | | | | | |
| 18-34 | 88 | 30.0 | 9.9 | 8.2 | 21.6% |
| 35-49 | 107 | 26.2 | 8.2 | 8.2 | 19.1% |
| 50-65 | 140 | 27.7 | 9.8 | 8.2 | 20.5% |
| **Women** | | | | | |
| 18-34 | 91 | 29.5 | 11.1 | 8.2 | 21.9% |
| 35-49 | 90 | 25.4 | 9.9 | 8.2 | 19.5% |
| 50-65 | 99 | 27.2 | 9.7 | 8.2 | 20.2% |

Values are percentage. A combined SD of 22% was used to classify participants in the present study.
BMI, body mass index; CVmTEE, within-individual variation in total energy expenditure; CVpER, within-indiviudal variation in predicted energy requirements; CVrEI, within-individual variation in self-reported energy intake; SD, standard deviation.

**Supplemental Table S4.** Median intakes of low nutritive value foods among 1147 French-speaking adults from 5 administrative regions of the Province of Quebec, Canada

| Sociodemographic characteristics | *n* (weighted) | Energy intake (kcal) from low nutritive value foods | | | Percentage of total energy intake (%E) from low nutritive value foods | | |
|---|---|---|---|---|---|---|---|
| | | Median | (95%CI) | [Q1 to Q3] | Median | (95%CI) | [Q1 to Q3] |
| **All** | | | | | | | |
| All | 1147 | 627 | (595-659) | [407 to 940] | 27.7 | (26.8-28.5) | [19.1 to 37.9] |
| **Sex** | | | | | | | |
| Men | 571 | 741 | (696-786) | [470 to 1093] | 28.6 | (27.4-29.8) | [20.6 to 39.1] |
| Women | 576 | 532 | (493-572) | [332 to 782] | 26.2 | (24.9-27.5) | [18.2 to 35.7] |
| **Age group, y** | | | | | | | |
| 18-34 | 408 | 678 | (614-741) | [426 to 983] | 29.6 | (27.6-31.6) | [19.8 to 39.1] |
| 35-49 | 338 | 609 | (557-661) | [400 to 948] | 26.2 | (24.5-28.0) | [18.7 to 36.5] |
| 50-65 | 400 | 592 | (549-635) | [382 to 878] | 27.7 | (26.4-29.0) | [19.1 to 36.5] |
| **Administrative region** | | | | | | | |
| Estrie | 110 | 595 | (502-688) | [400 to 912] | 26.8 | (23.6-30.1) | [19.3 to 36.5] |
| Saguenay-Lac-Saint-Jean | 107 | 682 | (594-769) | [443 to 994] | 28.0 | (24.9-31.2) | [19.7 to 42.4] |
| Capitale-Nationale / Chaudière-Appalaches | 435 | 653 | (604-702) | [415 to 949] | 28.4 | (26.8-30.0) | [19.6 to 38.2] |
| Montreal | 397 | 571 | (526-615) | [372 to 854] | 25.9 | (24.1-27.8) | [18.5 to 36.2] |
| Mauricie | 99 | 688 | (612-764) | [409 to 986] | 30.0 | (26.3-33.7) | [19.6 to 39.1] |
| **BMI group*** | | | | | | | |
| Normal (<25.0) | 403 | 578 | (531-625) | [379 to 877] | 26.8 | (25.2-28.3) | [18.5 to 35.9] |
| Overweight (25.0-29.9) | 344 | 664 | (605-722) | [429 to 952] | 28.3 | (26.8-29.8) | [20.5 to 37.9] |
| Obese (≥30.0) | 281 | 687 | (617-757) | [397 to 1026] | 28.4 | (26.3-30.5) | [18.9 to 39.6] |
| **Education*** | | | | | | | |
| High school or less | 269 | 650 | (595-704) | [400 to 989] | 29.1 | (27.5-30.6) | [19.9 to 39.1] |
| CEGEP † | 335 | 623 | (565-681) | [418 to 929] | 27.9 | (26.0-29.8) | [19.6 to 38.5] |
| University | 486 | 611 | (564-657) | [393 to 914] | 26.0 | (24.4-27.6) | [18.7 to 35.6] |
| **Household income*, $CAD** | | | | | | | |
| < 30, 000 | 162 | 577 | (488-666) | [337 to 888] | 26.3 | (23.5-29.2) | [18.7 to 38.1] |
| ≥ 30, 000 to < 60, 000 | 284 | 630 | (575-685) | [379 to 968] | 27.9 | (26.1-29.7) | [18.0 to 37.9] |
| ≥ 60, 000 to < 90, 000 | 196 | 583 | (505-661) | [408 to 885] | 26.0 | (23.6-28.4) | [18.3 to 36.9] |
| ≥ 90, 000 | 352 | 697 | (642-753) | [466 to 993] | 28.9 | (27.1-30.7) | [20.7 to 37.9] |

BMI, body mass index; CI, confidence intervals; d, day; Q1, lower quartile; Q3, upper quartile.
* The numbers may not sum to the total number of participants due to missing data. Variance estimation for quantiles using imputed data and the jackknife method is not supported in SAS®.
† CEGEP (*collège d'enseignement général et professionnel*) is a pre-university and technical college institution specific to the Quebec educational system.

**Supplemental Table S5.** "Naive" associations between incremental consumption of 250 calories from low nutritive value foods and cardiometabolic risk markers

| | Regression coefficients (95%CI) | | | |
|---|---|---|---|---|
| | **"Naive" model 1** | **"Naive" model 2** | **"Naive" model 3** | **"Naive" model 4** |
| | (basic) | (basic + adiposity) | (basic + adiposity + lifestyle) | (basic + adiposity + lifestyle + energy and diet quality) |
| **Anthropometric measures** | | | | |
| Waist circumference, cm | 2.6 (2.0 to 3.3) | 0.3 (0.0 to 0.5) | 0.2 (0.0 to 0.4) | -0.3 (-0.8 to 0.1) |
| *P* | <0.001 | 0.02 | 0.12 | 0.13 |
| BMI, kg/m$^2$ | 1.0 (0.8 to 1.3) | 1.1 (0.8 to 1.3) | 0.9 (0.7 to 1.2) | 0.0 (-0.6 to 0.5) |
| *P* | <0.001 | <0.001 | <0.001 | 0.89 |
| Body fat, % | 1.3 (1.0 to 1.6) | 0.2 (0.0 to 0.3) | 0.1 (0.0 to 0.3) | 0.1 (-0.1 to 0.3) |
| *P* | <0.001 | 0.07 | 0.18 | 0.39 |
| **Serum lipids, mmol/L** | | | | |
| Total cholesterol | 0.05 (0.01 to 0.09) | 0.06 (0.01 to 0.10) | 0.06 (0.01 to 0.10) | 0.06 (0.0 to 0.13) |
| *P* | 0.01 | 0.01 | 0.02 | 0.06 |
| LDL cholesterol | 0.04 (0.00 to 0.07) | 0.03 (0.00 to 0.07) | 0.03 (0.00 to 0.07) | 0.06 (0.01 to 0.12) |
| *P* | 0.04 | 0.09 | 0.07 | 0.02 |
| HDL cholesterol | -0.03 (-0.05 to -0.01) | -0.01 (-0.02 to 0.01) | 0.01 (-0.02 to 0.01) | -0.01 (-0.05 to 0.02) |
| *P* | 0.001 | 0.50 | 0.55 | 0.40 |
| TG*, % | 6.6 (4.4 to 8.9) | 4.1 (2.0 to 6.4) | 3.5 (1.3 to 5.7) | 3.4 (-0.1 to 7.0) |
| *P* | <0.001 | <0.001 | 0.001 | 0.06 |
| Non-HDL cholesterol | 0.08 (0.04 to 0.12) | 0.06 (0.02 to 0.10) | 0.06 (0.02 to 0.10) | 0.08 (0.02 to 0.14) |
| *P* | <0.001 | 0.004 | 0.005 | 0.02 |
| Cholesterol:HDL cholesterol | 0.12 (0.07 to 0.17) | 0.06 (0.01 to 0.11) | 0.06 (0.01 to 0.11) | 0.07 (-0.02 to 0.15) |
| *P* | <0.001 | 0.02 | 0.03 | 0.12 |
| **Blood pressure, mm Hg** | | | | |
| SBP | 0.9 (0.4 to 1.5) | 0.1 (-0.4 to 0.7) | 0.1 (-0.4 to 0.6) | 0.2 (-0.7 to 1.0) |
| *P* | 0.001 | 0.58 | 0.70 | 0.71 |
| DBP | 1.0 (0.5 to 1.4) | 0.3 (-0.1 to 0.8) | 0.3 (-0.1 to 0.7) | 0.4 (-0.3 to 1.1) |
| *P* | <0.001 | 0.10 | 0.13 | 0.26 |
| **Glucose homeostasis** | | | | |
| Fasting glucose*, % | 0.1 (-0.4 to 0.6) | -0.5 (-1.0 to 0.1) | -0.5 (-1.1 to 0.0) | -0.6 (-1.4 to 0.2) |
| *P* | 0.69 | 0.08 | 0.07 | 0.15 |
| Fasting insulin*, % | 4.5 (2.5 to 6.5) | 0.3 (-1.4 to 2.2) | 0.2 (-1.6 to 2.0) | 0.8 (-2.0 to 3.6) |
| *P* | <0.001 | 0.71 | 0.83 | 0.60 |
| HOMA-IR*, % | 4.6 (2.4 to 6.8) | -0.1 (-2.1 to 1.8) | -0.3 (-2.3 to 1.7) | 0.2 (-2.9 to 3.3) |
| *P* | <0.001 | 0.88 | 0.76 | 0.92 |
| **Inflammation** | | | | |
| C-reactive protein*, % | 15.7 (10.0 to 21.6) | 3.4 (-0.7 to 7.8) | 2.8 (-1.3 to 7.2) | 1.6 (-4.6 to 8.2) |
| *P* | <0.001 | 0.10 | 0.18 | 0.61 |

All values are regression coefficients (95% CI). Naive models do not account for within-person random error. P-values are the partial effect of calories consumed as low nutritive value foods in the linear models. Covariates are as follows, **model 1:** age, sex, center, number of weekend recalls, reporting status; **model 2:** model 1 and BMI (except for the outcome BMI), BMI-adjusted waist circumference (except for the outcome waist circumference and body fat percentage); **model 3:** model 2 and physical activity, smoking, dietary supplement usage, medication usage and education level; **model 4:** model 3 and total energy intake, diet quality (Canadian Healthy Eating Index 2007).

* Analyses were performed on log-transformed data. Hence, values are expressed as percentage change upon backtransformation calculated as 100 × exponential ($\log\beta x$) – 100

BMI, body mass index; DBP, diastolic blood pressure; CI, confidence intervals; HDL, high-density lipoproteins; LDL, low-density lipoproteins; SBP, systolic blood pressure; TG, triglycerides

**Supplemental Table S6.** "Naive" associations between incremental consumption of 10% of total energy intakes from low nutritive value foods and cardiometabolic risk markers

| | Regression coefficients (95%CI) | | |
|---|---|---|---|
| | **"Naive" model 1** | **"Naive" model 2** | **"Naive" model 3** |
| **Anthropometric measures** | | | |
| Waist circumference, cm | 1.7 (1.0 to 2.4) | 0.1 (-0.1 to 0.4) | 0.0 (-0.3 to 0.3) |
| *P* | <0.001 | 0.33 | 0.97 |
| BMI, kg/m$^2$ | 0.7 (0.4 to 1.0) | 0.7 (0.4 to 1.0) | 0.5 (0.2 to 0.8) |
| *P* | <0.001 | <0.001 | <0.001 |
| Body fat, % | 1.0 (0.7 to 1.4) | 0.3 (0.1 to 0.5) | 0.2 (0.0 to 0.4) |
| *P* | <0.001 | 0.002 | 0.01 |
| **Serum lipids, mmol/L** | | | |
| Total cholesterol | 0.08 (0.03 to 0.13) | 0.08 (0.03 to 0.13) | 0.08 (0.03 to 0.14) |
| *P* | 0.001 | 0.001 | 0.001 |
| LDL cholesterol | 0.06 (0.01 to 0.10) | 0.05 (0.01 to 0.10) | 0.06 (0.01 to 0.10) |
| *P* | 0.01 | 0.02 | 0.01 |
| HDL cholesterol | -0.02 (-0.04 to 0.0) | 0.0 (-0.02 to 0.02) | 0.0 (-0.02 to 0.02) |
| *P* | 0.088 | 0.69 | 0.75 |
| TG*, % | 6.5 (3.9 to 9.3) | 4.9 (2.3 to 7.5) | 4.0 (1.4 to 6.7) |
| *P* | <0.001 | <0.001 | 0.002 |
| Non-HDL cholesterol | 0.10 (0.05 to 0.15) | 0.09 (0.04 to 0.14) | 0.09 (0.04 to 0.14) |
| *P* | <0.001 | 0.001 | 0.001 |
| Cholesterol:HDL cholesterol | 0.12 (0.06 to 0.18) | 0.08 (0.02 to 0.14) | 0.08 (0.02 to 0.14) |
| *P* | <0.001 | 0.007 | 0.01 |
| **Blood pressure, mm Hg** | | | |
| SBP | 0.7 (0.1 to 1.3) | 0.2 (-0.4 to 0.8) | 0.1 (-0.5 to 0.7) |
| *P* | 0.03 | 0.53 | 0.68 |
| DBP | 0.9 (0.4 to 1.4) | 0.5 (0.0 to 1.0) | 0.4 (0.0 to 0.9) |
| *P* | <0.001 | 0.04 | 0.06 |
| **Glucose homeostasis** | | | |
| Fasting glucose*, % | 0.0 (-0.6 to 0.6) | -0.4 (-1.0 to 0.3) | -0.4 (-1.1 to 0.3) |
| *P* | 0.98 | 0.29 | 0.28 |
| Fasting insulin*, % | 3.5 (1.1 to 6.1) | 0.9 (-1.3 to 3.2) | 0.8 (-1.5 to 3.1) |
| *P* | 0.004 | 0.41 | 0.50 |
| HOMA-IR*, % | 3.5 (0.9 to 6.3) | 0.6 (-1.8 to 3.0) | 0.4 (-2.0 to 2.9) |
| *P* | 0.01 | 0.63 | 0.74 |
| **Inflammation** | | | |
| C-reactive protein*, % | 13.0 (6.8 to 19.4) | 5.0 (0.1 to 10.0) | 4.2 (-0.7 to 9.3) |
| *P* | <0.001 | 0.04 | 0.10 |

All values are regression coefficients (95% CI). Naive models do not account for within-person random error. P-values are the partial effect of calories consumed as low nutritive value foods in the linear models. Covariates are as follows, **model 1:** age, sex, center, number of weekend recalls, reporting status; **model 2:** model 1 and BMI (except for the outcome BMI), BMI-adjusted waist circumference (except for the outcome waist circumference and body fat percentage); **model 3:** model 2 and physical activity, smoking, dietary supplement usage, medication usage and education level.

* Analyses were performed on log-transformed data. Hence, values are expressed as percentage change upon backtransformation calculated as 100 × exponential (log$\beta$x) – 100

BMI, body mass index; DBP, diastolic blood pressure; CI, confidence intervals; HDL, high-density lipoproteins; LDL, low-density lipoproteins; SBP, systolic blood pressure; TG, triglycerides

**Supplemental results**

**Supplemental Table S7.** Associations between incremental consumption of 250 calories from low nutritive value foods and cardiometabolic risk markers, stratified by median Canadian Healthy Eating Index scores

| | Regression coefficients (95%CI) | |
|---|---|---|
| | **Low C-HEI** | **High C-HEI** |
| | **Model 3** | **Model 3** |
| **Anthropometric measures** | | |
| Waist circumference, cm | 1.0 (0.2 to 1.9) | 0.2 (-0.5 to 0.9) |
| *P* | 0.02 | 0.59 |
| BMI, kg/m$^2$ | 2.8 (2.0 to 3.6) | 1.0 (0.3 to 1.8) |
| *P* | <0.001 | 0.005 |
| Body fat, % | 0.3 (-0.3 to 0.8) | -0.4 (-0.9 to 0.1) |
| *P* | 0.33 | 0.12 |
| **Serum lipids, mmol/L** | | |
| Total cholesterol | 0.05 (-0.12 to 0.21) | 0.12 (-0.02 to 0.26) |
| *P* | 0.60 | 0.10 |
| LDL cholesterol | -0.02 (-0.15 to 0.12) | 0.12 (-0.01 to 0.25) |
| *P* | 0.80 | 0.07 |
| HDL cholesterol | 0.0 (-0.07 to 0.07) | -0.03 (-0.08 to 0.03) |
| *P* | 0.99 | 0.37 |
| TG*, % | 7.5 (-0.8 to 16.5) | 4.6 (-1.6 to 11.2) |
| *P* | 0.08 | 0.15 |
| Non-HDL cholesterol | 0.04 (-0.12 to 0.21) | 0.14 (0.01 to 0.28) |
| *P* | 0.59 | 0.04 |
| Cholesterol:HDL cholesterol | 0.06 (-0.14 to 0.26) | 0.13 (-0.03 to 0.28) |
| *P* | 0.55 | 0.12 |
| **Blood pressure, mm Hg** | | |
| SBP | 0.9 (-1.0 to 2.8) | -0.8 (-2.3 to 0.8) |
| *P* | 0.34 | 0.32 |
| DBP | 0.6 (-1.0 to 2.2) | -0.7 (-1.8 to 0.3) |
| *P* | 0.47 | 0.18 |
| **Glucose homeostasis** | | |
| Fasting glucose*, % | -0.5 (-2.3 to 1.3) | -2.4 (-4.1 to -0.6) |
| *P* | 0.57 | 0.01 |
| Fasting insulin*, % | -3.8 (-10.5 to 3.5) | -2.8 (-8.6 to 3.4) |
| *P* | 0.30 | 0.37 |
| HOMA-IR*, % | -4.2 (-11.6 to 3.7) | -5.1 (-11.4 to 1.6) |
| *P* | 0.28 | 0.13 |
| **Inflammation** | | |
| C-reactive protein*, % | -5.2 (-18.4 to 10.1) | 5.5 (-7.7 to 20.6) |
| *P* | 0.48 | 0.43 |

All values are regression coefficients (95% CI). P-values are the partial effect of calories from low nutritive value foods in the linear models. Usual intakes were computed using the NCI methods 2.1 and one-part models. Covariates are as follows: age, sex, center, number of weekend recalls, reporting status, BMI (except for the outcome BMI), BMI-adjusted waist circumference (except for the outcome waist circumference and body fat percentage), physical activity, smoking, dietary supplement usage, medication usage and education level. Participants were dichotomized according to the sex-specific median of the Canadian Healthy Eating Index score which was 51.1 and 57.9 in men and women, respectively.

* Analyses were performed on log-transformed data. Hence, values are expressed as percentage change upon backtransformation calculated as 100 × exponential (log$\beta$x) – 100

BMI, body mass index; DBP, diastolic blood pressure; C-HEI, Canadian Healthy Eating Index; CI, confidence intervals; HDL, high-density lipoproteins; LDL, low-density lipoproteins; SBP, systolic blood pressure; TG, triglycerides

**Supplemental results**

**Supplemental Figure S1.** Proportion of total energy intake provided by the main sources of low nutritive value foods according to sociodemographic characteristics.

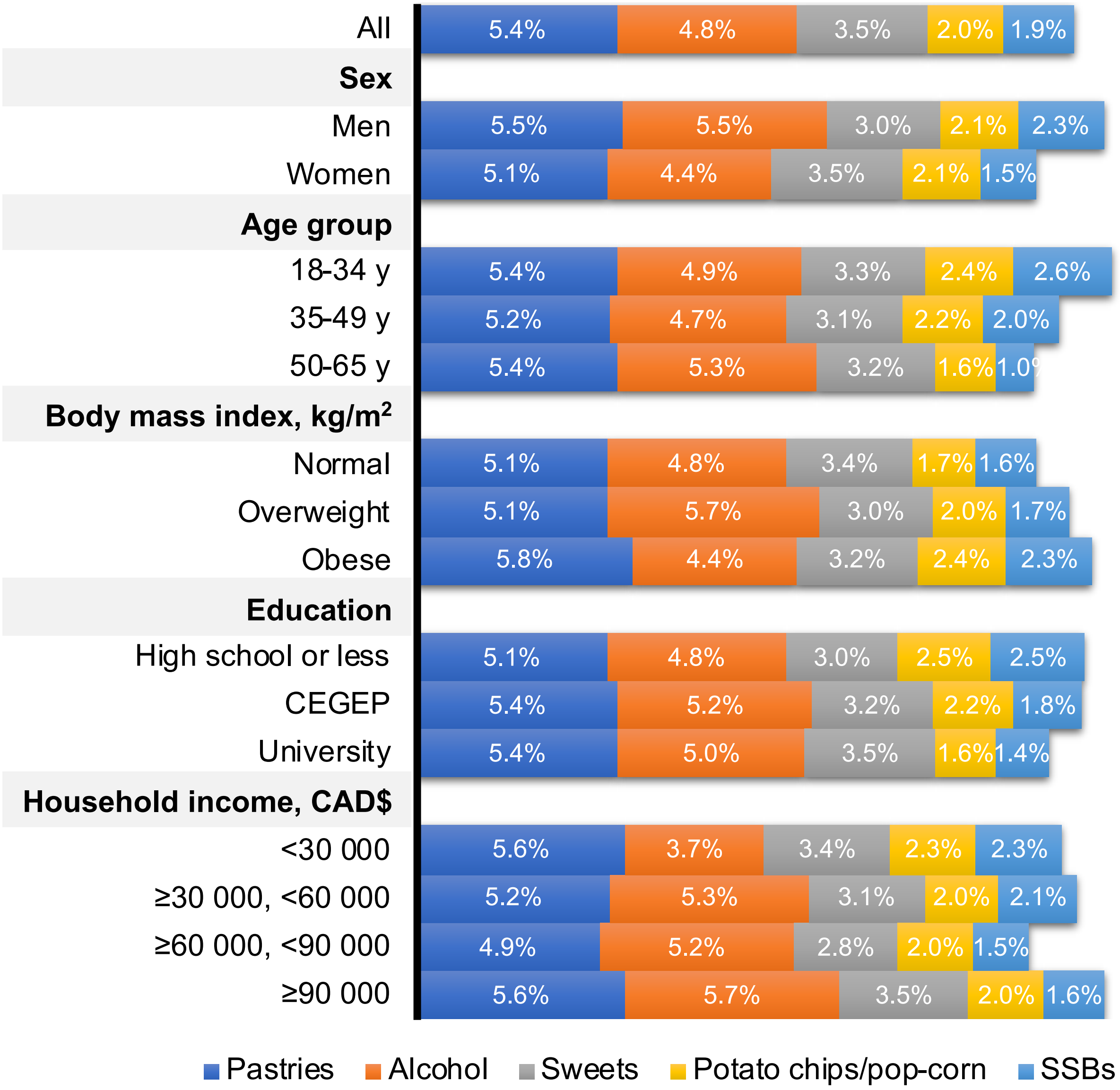

## Supplemental references


1. Health Canada. The development and use of a surveillance tool the classification of foods in the Canadian nutrient file according to Eating well with Canada's food guide. Ottawa 2014 [1-25]. Available from: http://publications.gc.ca/collections/collection_2014/sc-hc/H164-158-2-2014-eng.pdf. Accessed on June 2019.
2. Huang TT, Roberts SB, Howarth NC, McCrory MA. Effect of screening out implausible energy intake reports on relationships between diet and BMI. Obes Res. 2005;13(7):1205-17.
3. Otten JJ, Hellwig JP, Meyers LD. Dietary reference intakes : the essential guide to nutrient requirements. Washington, D.C.: National Academies Press; 2006. xiii, 543 p.
4. Black AE, Cole TJ. Within- and between-subject variation in energy expenditure measured by the doubly-labelled water technique: implications for validating reported dietary energy intake. Eur J Clin Nutr. 2000;54(5):386-94.
